# Interleukin-1 Receptor Blockade in Perinatal Brain Injury

**DOI:** 10.3389/fped.2014.00108

**Published:** 2014-10-07

**Authors:** Jason M. Rosenzweig, Jun Lei, Irina Burd

**Affiliations:** ^1^Department of Gynecology and Obstetrics, Integrated Research Center for Fetal Medicine, Johns Hopkins University School of Medicine, Baltimore, MD, USA; ^2^Department of Neuroscience, Kennedy Krieger Institute, Baltimore, MD, USA; ^3^Department of Neurology, Johns Hopkins University School of Medicine, Baltimore, MD, USA

**Keywords:** IL-1, rIL-RA, perinatal brain injury, IL-1beta, Kineret

## Abstract

Interleukin-1 (IL-1) is a potent inflammatory cytokine that can be produced by a variety of cell types throughout the body. While IL-1 is a central mediator of inflammation and response to infection, the role of IL-1 signaling in adult and pediatric brain injury is becoming increasingly clear. Although the mechanisms of IL-1 expression are largely understood, the downstream effects and contributions to excitotoxicity and oxidative stress are poorly defined. Here, we present a review of mechanisms of IL-1 signaling with a focus on the role of IL-1 in perinatal brain injury. We highlight research models of perinatal brain injury and the use of interleukin-1 receptor antagonist (IL-1RA) as an agent of therapeutic potential in preventing perinatal brain injury due to exposure to inflammation.

## Introduction

Interleukin-1 (IL-1) is the name given to two cytokine peptides, IL-1α and IL-1β, that bind and activate the IL-1 receptor (IL-1R). IL-1 was first called endogenous pyrogen and described as a protein isolated from polymorphonuclear leukocytes, that, when injected into humans or animals, causes fever (1, 2). IL-1 is a pro-inflammatory cytokine that mediates the immune response to infection and inflammation and influences a broad range of physiologic activity that includes acute-phase response gene expression, T and B lymphocyte stimulation, cell survival, glial activation, fever, hypotension, and leukopenia (3–6).

Mounting evidence suggests that IL-1 signaling plays a central role in mediating chronic and acute brain injury in both adult and pediatric populations. IL-1 receptor antagonist (IL-1RA) is an endogenous inhibitor of IL-1 signaling and recombinant IL-1RA is widely used in adults for treatment of autoimmune and inflammatory diseases such as rheumatoid arthritis and inflammatory bowel disease (5). There is emerging evidence that perinatal administration of IL-1RA may confer neuroprotective effects during births at high risk for brain injury (7, 8). Here, we offer a review of IL-1 and the therapeutic potential of IL-1RA in preventing brain injury in neonates following exposure to inflammation [either intrauterine inflammation preceding preterm birth, chorioamnionitis, or birth following hypoxia–ischemia (HI)].

## IL-1 Receptor

The IL-1R is comprised of two membrane proteins, IL-1R1 and IL-1R accessory protein (IL-1RAcP), and binds IL-1α, IL-1β, IL-1RA, and IL-38. IL-1R1 and IL-1RAcP contain an intracellular Toll/interleukin-1 receptor (TIR) homology domain that recruits myeloid differentiation primary response protein 88 (MyD88) upon receptor heterodimerization (4, 9). MyD88, an adapter protein, recruits the IL-1R associated kinase 4 (IRAK4), which initiates a signaling cascade by phosphorylating and activating IRAK1, which, in turn, activates and recruits TRAF6 to the IL-1R complex. TRAF6 mediates signaling through two pathways. One pathway leads to the activation of the transcription factor NFκB through the activation of TAB2 and TAK1. The other pathway leads to the activation of the c-jun/c-fos heterodimeric activating protein 1 (AP-1) transcription factor complex through the MAPK/JNK pathway. NFκB and AP-1 activation drives expression of the pro-inflammatory genes TNF-α, IL-6, and IL-1, generating an acute-phase response (10, 11).

A decoy receptor made up of IL-1R2 and IL-1RAcP also binds IL-1β but IL-1R2 lacks an intracellular activation domain (12). IL-1α and IL-1β bind IL-1R2 with much greater affinity than IL-1R1. The difference in affinity for the decoy receptor effectively creates a cytokine trap that neutralizes free IL-1 without inflammatory signaling (13). Additionally, an endogenous receptor antagonist, discussed in greater detail below, has a much lower affinity for IL-R2. Together, the decoy receptor and antagonist provide a potent mechanism of regulating IL-1 signaling.

## IL-1α and IL-1β

Both IL-1α and IL-1β transcripts are translated into precursor peptides. However, while pro-IL-1β requires proteolytic cleavage to generate the mature, active form of the cytokine, pro-IL-α is functionally active. Thus, IL-1α, which is expressed in epithelial cells of the gastrointestinal tract, kidney, liver, lung, endothelial cells, and astrocytes, rapidly initiates inflammatory responses when released by necrotic cell death, as occurs following ischemic events. IL-1β is expressed in hematopoietic cells, including macrophages, dendritic cells, monocytes, and microglia, and in endothelial cells. Expression of IL-1β is triggered by Toll-like receptor 4 (TLR4) signaling, IL-6 signaling, or by IL-1β itself. As many cell types express the IL-1R, IL-1 signaling can be paracrine or autocrine. The production and release of IL-1β is highly regulated by cells. TLR signaling is required for the expression and translation of pro-IL-1β. Maturation of IL-1β requires cleavage of pro-IL-1β by Caspase 1. Caspase 1 activity is regulated by the NLRP3 inflammasome. The inflammasome can be activated by numerous stimuli, including pathogen associate molecular patterns (PAMPS), external ATP and glucose, and molecules that signal of stress or danger (14, 15).

## IL-1 Receptor Antagonist

**Figure 1:**
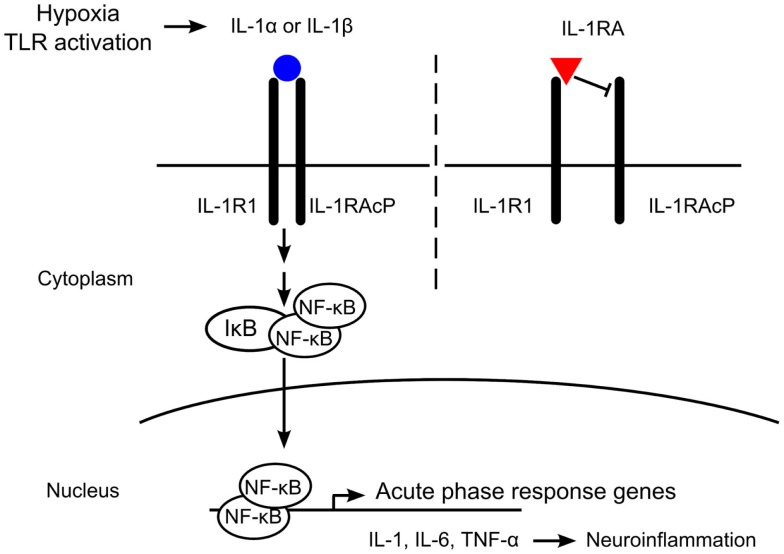
**Mechanism of interleukin-1 (IL-1) receptor antagonist (IL-1RA) blockade**. IL-1 is produced in response to hypoxia or Toll-like receptor (TLR) activation. The IL-1 receptor (IL-1R) is comprised of an IL-1R1 subunit and an IL-1R accessory protein (IL-1RAcP). IL-1RA binds IL-1R1 with a higher affinity than IL-1α or IL-1β, but does not recruit IL-1RAcP. Without heterodimerization of the IL-1 receptor complex, no signaling occurs. Binding of IL-1α or IL-1β to IL-1R1 recruits IL-1RAcP and intracellular signaling is initiated, leading to the expression of acute-phase response genes such as IL-1, interleukin-6 (IL-6), and tumor necrosis factor-alpha (TNF-α). In the brain, these pro-inflammatory cytokines induce neuroinflammation, including neuronal injury and astrogliosis.

The IL-1 receptor antagonist (IL-1RA) is an endogenous ligand that binds the IL-1R but does not recruit the IL-1RAcP, thereby preventing activation of the receptor (Figure 1). IL-1RA also has a higher affinity for the IL-1R than IL-1α or IL-1β and serves to limit pro-inflammatory IL-1 signaling by blocking binding of the active cytokines (16). Deficiencies in IL-1RA result in a reduction of regulatory function and can result in severe inflammation and autoinflammatory disorders such as arthritis, vasculitis, and skin lesions in humans (17–19). IL-1RA knockout mice develop similar phenotypes to those seen in human disease, including arthropathy and arterial inflammation (20–22).

IL-1RA, IL-1α, and IL-1β have been shown to cross the blood–brain barrier by a saturable mechanism (23). In rat models of stroke, rIL-RA, delivered subcutaneously or intravenously, can reach therapeutic concentrations in the cerebrospinal fluid within 45 min (24, 25). While the placenta can secrete IL-RA in response to lipopolysaccharide, to date, no studies have addressed placental transfer of rIL-1RA (26). Placental perfusion studies have found that several cytokines, including IL-1β, TNF-α, and IL-6, do not cross the placenta and this may hold true for rIL-1RA (27–29). Pharmacokinetic studies are warranted to determine the potential efficacy of maternally administered rIL-1RA in the setting of preterm birth. In considering possible future clinical applications, rIL-1RA may need to be delivered via amniocentesis if placental transfer is insufficient to generate therapeutic doses in the fetal compartment. With future advances and knowledge of molecular action of IL-1RA, we speculate that small molecules may be designed that will mimic IL-1RA activity and would be able to cross the placenta.

## Clinical Trials with IL-1RA

In 1993, Amgen introduced the first drug targeting IL-1 signaling, a recombinant IL-1RA (rIL-1RA), Anakinra (Kineret). rIL-1RA is produced in *E. coli* and was approved by the FDA for treatment of rheumatoid arthritis in 2001. In 2012, rIL-RA was approved for treatment of neonatal-onset multisystem inflammatory disease (NOMID). Five randomized, double-blind, placebo-controlled trials that included over 3000 patients were conducted (30–34). Mertens and Singh offer a critical review of the rIL-1RA clinical trials (35). Briefly, the trials found rIL-1RA to be significantly more effective than placebo in improving outcomes with no difference in adverse events, deaths, and study withdrawals.

Interest in recombinant rIL-1RA therapy for additional diseases continues, and at this time there are 21 ongoing clinical trials to treat a range of diseases including diabetes, breast cancer, chronic fatigue, and heart failure (Table 1). Recent mechanistic studies have reported that rIL-1RA has neuroprotective effects in rodent models of perinatal brain injury (7, 8, 24, 25).

**Table 1:** **Ongoing rIL-1RA clinical trials**.

Study title	Phase	Primary outcome measures	Anakinra dose
Anakinra combined with chemotherapy and dendritic cell vaccine to treat breast cancer	1/2	Safety of DC vaccine combined with chemotherapy, and DC vaccine combined with chemotherapy and anakinra	100 mg/day subcutaneous
Infants and children with coronary artery abnormalities in acute Kawasaki disease	1/2	Safety of a 6-week course of anakinra	2 mg/kg/day 4 mg/kg/day
Adult patients with colchicine-resistant familial Mediterranean fever	3	Number of patients with less than a mean of one FMF attack per month	100 mg/day subcutaneous
Safety and blood immune cell study of anakinra in metastatic breast cancer patients	1	Safety – adverse events in participants	100 mg/day subcutaneous
Anakinra or denosumab and everolimus in advanced cancer	1	Maximum tolerated dose (MTD)	100 mg/day subcutaneous
Efficacy study of anakinra, pentoxifylline, and zinc compared to methylprednisolone in severe acute alcoholic hepatitis	2/3	Death|MELD score	100 mg/day subcutaneous
Safety and tolerability of anakinra in combination with riluzol in amyotrophic lateral sclerosis	2	Number and severity of adverse events, pathological laboratory parameters	100 mg/day subcutaneous
IL-1 blockade in acute myocardial infarction (VCU-ART3)	2/3	Acute response (CRP levels)	100 mg/day subcutaneous
Study evaluating the influence of LV5FU2 bevacizumab plus anakinra association on metastatic colorectal cancer	2	Response rate after 2 months in patients with colorectal cancer with liver metastases treated with anakinra and LV5FU2/bevacizumab	100 mg/day subcutaneous
Evaluation of the safety of anakinra plus standard chemotherapy	1	The number of participants with serious adverse events and adverse events	100 mg/day subcutaneous
IL-1 blockade in acute heart failure (anakinra ADHF)	2/3	C reactive protein	200 mg/day for 3 days (high dose)
			100 mg/day (standard dose)
Interleukin-1 blockade in recently decompensated heart failure	2/3	Placebo-corrected interval changes in peak VO2 and VE/VCO2 slope	100 mg/day subcutaneous
Inflammatory pustular skin diseases	2	Obtain an estimate of the response rate to treatment	100–300 mg/day subcutaneous
Effect of anakinra on insulin sensitivity in type 1 diabetes mellitus	2	Insulin sensitivity as determined by euglycemic hyperinsulinemic clamp	100 mg/day subcutaneous
Gene expression profiling in PBMCs as a tool for prediction of anakinra responsiveness in rheumatoid arthritis	4	Observational	100 mg/day subcutaneous
Role of interleukin-1 in postprandial fatigue	1	Postprandial fatigue	100 mg subcutaneous
Immunomodulation, IL-1 inhibition, and postoperative incisional pain	N/A^a^	Concentration levels of inflammatory mediators (IL-1, IL-6, IL-8, and TNF-α) present in human wounds following surgery with and without the use of anakinra	N/A^a^
Cytokine inhibition in chronic fatigue syndrome patients	2/3	CIS (checklist individual strength, compared to baseline)	100 mg/day subcutaneous
A dose-block randomized, placebo controlled (double-blind), active controlled(open-label), dose-escalation study	1	Tolerability, pharmacokinetics of HL2351, Immunogenicity of HL2351, Tolerability, pharmacokinetics, and pharmacodynamics of HL2351 in comparison with kineret (anakinra), IL-6 inhibition assay	100 mg/day subcutaneous
Anti-IL-1 treatment in children DKA at diagnosis of type 1 diabetes	2	Number of adverse events	2 mg/kg bolus followed by 2 mg/kg/h infusion
Interleukin-1 blockade in HF with preserved EF	2	Aerobic exercise capacity, ventilatory efficiency	100 mg/day subcutaneous

*^a^Data not available*.

## IL-1RA in Brain Injury

Many antenatal, perinatal, or postnatal factors, whether genetic or environmental, can lead to postnatal brain injury. Chronic events prior to parturition may be of greater importance than acute events, as the chronic conditions may be overlooked until postnatal clinical symptoms are evident. Additionally, the timing of the insult influences the nature of the brain injury. Preterm infants are more likely to suffer from intraventricular hemorrhage and periventricular leukomalacia, while term infants experience focal ischemia, injury to basal ganglia, and subcortical hemorrhage (36).

Pathogen-induced inflammation and/or HI are the major insults resulting in postnatal neurological impairment through the release of inflammatory mediators, such as members of the IL-1 family (37–43). Human neuropathological studies and experimental animal models of postnatal brain injury reveal that pro-inflammatory cytokines, especially IL-1β, are implicated in the cascade leading to brain damage at different developmental stages (41, 44–50). Therefore, postnatal systemic administration of IL-1RA may be a potential therapeutic intervention of postnatal brain injury (7, 51).

## IL-1RA and Pathogenic Models of Postnatal Brain Injury

Several models of inflammatory postnatal brain injury have been developed in different species, including mouse (52–56), rat (57–59), rabbit (60, 61), dog (62), and others (63). Rodent models are most widely used due to ease of use and relatively short reproductive cycle. Administration of pathogens, such as virus (52), bacteria (64), pathogenic infectious components (57, 61), and pro-inflammatory cytokines (46, 65) varies widely between models in timing, from prenatal to postnatal stage, and route of delivery, whether intranasal (52, 66), intravenous (67), intrauterine (68, 69), intraperitoneal (59), or intracerebral injection (70).

IL-1RA has been shown to be neuroprotective (71–73) in animal models of traumatic brain injury and excitotoxicity *in vivo* and *in vitro*, in which IL-1β exerts a dominant role pathologically. In rodent models of postnatal brain injury, the elevation of IL-1β and other pro-inflammatory cytokines was observed (69, 74, 75), indicating the importance of the IL-1β signaling pathway in postnatal brain injury. Leitner et al. applied rIL-1RA systemically at embryonic day 15, 30 min prior to administration of intrauterine injection of lipopolysaccharide. They found rIL-1RA improved fetal cortical neuronal injury without affecting the rate of preterm birth. This might be via the blockade of neuronal nitric oxide synthase (8). Furthermore, Girard et al. administrated a low dose of rIL-1RA to pups in a systemic inflammatory animal model and a hypoxic-ischemia model postnatally (7, 76). This treatment preserved motor function and exploratory behavior. Neuroprotective effects were evident by increased neural stem cell populations, prevention of myelin loss, and decreased gliosis. This study provides a potential candidate for postnatal treatment of brain injury, especially in the earliest days of life in the term infant. Savard et al. used systemic infection–inflammation combined with HI in a rat model at postnatal day 12, which exerted a synergistic detrimental effect on rat brain, leading to a peculiar pattern of parasagittal cortical–subcortical infarcts mimicking those in the human full-term newborn with subsequent severe neurodevelopmental impairments. rIL-1RA administration reduced the extent of brain lesions by MRI observation (50).

## IL-1RA and Hypoxia–Ischemia Associated Postnatal Brain Injury Model

Hypoxia–ischemia is another common cause of postnatal brain injury. The most widely used HI model is the Vannucci model, which combines permanent unilateral ligation of the carotid artery in 7-day-old rat pups, along with exposure to hypoxia (77–80). Increased expression of pro-inflammatory cytokines including IL-1β is associated with HI-induced postnatal brain injury (81–83).

Experimental administration of rIL-1RA has been demonstrated to reduce HI-induced postnatal brain injury (84–86). Martin et al. injected rIL-1RA subcutaneously in a postnatal rat HI model and found prior to or after HI, rIL-1RA ameliorated the ischemia damage as measured by hemisphere dry weights and prevented neuronal loss in the striatum (87). Hu et al. injected 2 μg rIL-1RA intra-cerebroventricularly 2 h after HI and found a significant reduction in cell death and Caspase 3 activity. The observed increase in cytoplasmic NFκB activation and nuclear translocation of Bcl-3 24 h after HI was also significantly attenuated by IL-1 blockade, suggesting that HI-induced IL-1 activation is via both the NFκB activation and the nuclear translocation of Bcl-3 (88).

Though a rapidly expanding body of evidence indicates that rIL-1RA is a promising therapeutic for postnatal brain injury, the specific signaling mechanisms triggered by rIL-1RA responsible for the effects are still not fully known. A number of drawbacks of rIL-1RA limit its broader use; these include injection site reactions (89, 90), broad immunosuppression (90), and high costs. Trials to test safety in a pediatric population are sorely needed as a lack of efficacy and safety data limits the adoption of rIL-1RA for perinatal brain injury.

## Concluding Remarks

During infection and inflammation, the potent effects of IL-1 signaling can lead to devastating tissue damage with long-lasting effects. Therapies that block IL-1 signaling have been successful in reducing negative outcomes in autoinflammatory diseases in adults for over a decade now. Exciting research in the area of neonatal encephalopathy suggests that the benefits of IL-1 blockade in reducing injury in autoinflammatory diseases may be extended to neonatal brain injury and offer much needed neuroprotection for a population with limited effective treatment options.

Neonatal encephalopathy affects up to 1% of live births (91–93) and the causes can vary from hypoxic–ischemic events to intrauterine inflammation (37, 38, 40–43). Treatment options are limited and the current standard of care prescribes therapeutic hypothermia (94). Hypothermia, however, does not confer complete neuroprotection and as many as 50% of treated neonates will experience moderate to severe neurologic disability (95). Common processes that contribute to neuronal injury, including oxidative stress, apoptosis, inflammation, and excitotoxicity, are increasingly the targets of emerging therapies for neonatal encephalopathy.

As a Class B drug, rIL-1RA is approved for use in pregnant women and may be offered in the future as a perinatal intervention to prevent perinatal brain injury due to neonatal encephalopathy or due to exposure to intrauterine inflammation. However, rIL-1RA is not approved for the treatment or prevention of perinatal brain injury, and further studies are needed to determine its safety and efficacy. At this time, little is known in regards to the importance of IL-1 in brain formation or the development of the immature immune system; therefore, further evaluation of this molecule is necessary to establish appropriate safe timing of its administration for variety of etiologies of perinatal brain injury. Furthermore, no studies have yet been conducted to assess the efficacy of rIL-1RA in combination with other therapies, such as hypothermia, although other combination therapies (hypothermia and erythropoietin) have shown promise in rodent models (96, 97). Mechanistic studies of r-IL1RA, as we are conducting, are ongoing to evaluate maternal–fetal transfer and developmental effects in animal models.

## Conflict of Interest Statement

The authors declare that the research was conducted in the absence of any commercial or financial relationships that could be construed as a potential conflict of interest.

## References

[1] Beeson PB Temperature-elevating effect of a substance obtained from polymorphonuclear leucocytes. J Clin Invest (1948) 27(4):524 18939147

[2] Atkins E Pathogenesis of fever. Physiol Rev (1960) 40:580–646 13794961 10.1152/physrev.1960.40.3.580

[3] Dinarello CA Interleukin-1 and interleukin-1 antagonism. Blood (1991) 77(8):1627–52 1826616

[4] Sims JE Smith DE The IL-1 family: regulators of immunity. Nat Rev Immunol (2010) 10(2):89–102 10.1038/nri2691 20081871

[5] Dinarello CA Simon A van der Meer JW Treating inflammation by blocking interleukin-1 in a broad spectrum of diseases. Nat Rev Drug Discov (2012) 11(8):633–52 10.1038/nrd3800 22850787 PMC3644509

[6] Garlanda C Dinarello CA Mantovani A The interleukin-1 family: back to the future. Immunity (2013) 39(6):1003–18 10.1016/j.immuni.2013.11.010 24332029 PMC3933951

[7] Girard S Sebire H Brochu ME Briota S Sarret P Sebire G Postnatal administration of IL-1Ra exerts neuroprotective effects following perinatal inflammation and/or hypoxic-ischemic injuries. Brain Behav Immun (2012) 26(8):1331–9 10.1016/j.bbi.2012.09.001 22982341 PMC5023428

[8] Leitner K Al Shammary M McLane M Johnston MV Elovitz MA Burd I IL-1 receptor blockade prevents fetal cortical brain injury but not preterm birth in a mouse model of inflammation-induced preterm birth and perinatal brain injury. Am J Reprod Immunol (2014) 71(5):418–26 10.1111/aji.12216 24592965 PMC3989434

[9] Dunne A O’Neill LA The interleukin-1 receptor/Toll-like receptor superfamily: signal transduction during inflammation and host defense. Sci STKE (2003) 2003(171):re3 10.1126/stke.2003.171.re3 12606705

[10] Watters TM Kenny EF O’Neill LA Structure, function and regulation of the Toll/IL-1 receptor adaptor proteins. Immunol Cell Biol (2007) 85(6):411–9 10.1038/sj.icb.7100095 17667936

[11] Casanova JL Abel L Quintana-Murci L Human TLRs and IL-1Rs in host defense: natural insights from evolutionary, epidemiological, and clinical genetics. Annu Rev Immunol (2011) 29:447–91 10.1146/annurev-immunol-030409-101335 21219179

[12] McMahan CJ Slack JL Mosley B Cosman D Lupton SD Brunton LL A novel IL-1 receptor, cloned from B cells by mammalian expression, is expressed in many cell types. EMBO J (1991) 10(10):2821–32 1833184 10.1002/j.1460-2075.1991.tb07831.x PMC452992

[13] Hannum CH Wilcox CJ Arend WP Joslin FG Dripps DJ Heimdal PL Interleukin-1 receptor antagonist activity of a human interleukin-1 inhibitor. Nature (1990) 343(6256):336–40 10.1038/343336a0 2137200

[14] Mariathasan S Newton K Monack DM Vucic D French DM Lee WP Differential activation of the inflammasome by caspase-1 adaptors ASC and Ipaf. Nature (2004) 430(6996):213–8 10.1038/nature02664 15190255

[15] Tschopp J Schroder K NLRP3 inflammasome activation: the convergence of multiple signalling pathways on ROS production? Nat Rev Immunol (2010) 10(3):210–5 10.1038/nri2725 20168318

[16] Dinarello CA Anti-inflammatory agents: present and future. Cell (2010) 140(6):935–50 10.1016/j.cell.2010.02.043 20303881 PMC3752337

[17] Aksentijevich I Masters SL Ferguson PJ Dancey P Frenkel J van Royen-Kerkhoff A An autoinflammatory disease with deficiency of the interleukin-1-receptor antagonist. N Engl J Med (2009) 360(23):2426–37 10.1056/NEJMoa0807865 19494218 PMC2876877

[18] Gabay C Palmer G Mutations in the IL1RN locus lead to autoinflammation. Nat Rev Rheumatol (2009) 5(9):480–2 10.1038/nrrheum.2009.177 19710672

[19] Reddy S Jia S Geoffrey R Lorier R Suchi M Broeckel U An autoinflammatory disease due to homozygous deletion of the IL1RN locus. N Engl J Med (2009) 360(23):2438–44 10.1056/NEJMoa0809568 19494219 PMC2803085

[20] Horai R Asano M Sudo K Kanuka H Suzuki M Nishihara M Production of mice deficient in genes for interleukin (IL)-1alpha, IL-1beta, IL-1alpha/beta, and IL-1 receptor antagonist shows that IL-1beta is crucial in turpentine-induced fever development and glucocorticoid secretion. J Exp Med (1998) 187(9):1463–75 10.1084/jem.187.9.1463 9565638 PMC2212263

[21] Horai R Saijo S Tanioka H Nakae S Sudo K Okahara A Development of chronic inflammatory arthropathy resembling rheumatoid arthritis in interleukin 1 receptor antagonist-deficient mice. J Exp Med (2000) 191(2):313–20 10.1084/jem.191.2.313 10637275 PMC2195765

[22] Nicklin MJ Hughes DE Barton JL Ure JM Duff GW Arterial inflammation in mice lacking the interleukin 1 receptor antagonist gene. J Exp Med (2000) 191(2):303–12 10.1084/jem.191.2.303 10637274 PMC2195758

[23] Banks WA Kastin AJ Broadwell RD Passage of cytokines across the blood-brain barrier. Neuroimmunomodulation (1995) 2(4):241–8 10.1159/000096887 8963753

[24] Greenhalgh AD Galea J Denes A Tyrrell PJ Rothwell NJ Rapid brain penetration of interleukin-1 receptor antagonist in rat cerebral ischaemia: pharmacokinetics, distribution, protection. Br J Pharmacol (2010) 160(1):153–9 10.1111/j.1476-5381.2010.00684.x 20412072 PMC2860215

[25] Galea J Ogungbenro K Hulme S Greenhalgh A Aarons L Scarth S Intravenous anakinra can achieve experimentally effective concentrations in the central nervous system within a therapeutic time window: results of a dose-ranging study. J Cereb Blood Flow Metab (2011) 31(2):439–47 10.1038/jcbfm.2010.103 20628399 PMC3049499

[26] Amash A Holcberg G Sapir O Huleihel M Placental secretion of interleukin-1 and interleukin-1 receptor antagonist in preeclampsia: effect of magnesium sulfate. J Interferon Cytokine Res (2012) 32(9):432–41 10.1089/jir.2012.0013 22909148 PMC3438822

[27] Reisenberger K Egarter C Vogl S Sternberger B Kiss H Husslein P The transfer of interleukin-8 across the human placenta perfused in vitro. Obstet Gynecol (1996) 87(4):613–6 10.1016/0029-7844(95)00473-4 8602318

[28] Zaretsky MV Alexander JM Byrd W Bawdon RE Transfer of inflammatory cytokines across the placenta. Obstet Gynecol (2004) 103(3):546–50 10.1097/01.AOG.0000114980.40445.83 14990420

[29] Aaltonen R Heikkinen T Hakala K Laine K Alanen A Transfer of proinflammatory cytokines across term placenta. Obstet Gynecol (2005) 106(4):802–7 10.1097/01.AOG.0000178750.84837.ed 16199639

[30] Bresnihan B Alvaro-Gracia JM Cobby M Doherty M Domljan Z Emery P Treatment of rheumatoid arthritis with recombinant human interleukin-1 receptor antagonist. Arthritis Rheum (1998) 41(12):2196–204 10.1002/1529-0131(199812)41:12<2196::AID-ART15>3.3.CO;2-U 9870876

[31] Cohen S Hurd E Cush J Schiff M Weinblatt ME Moreland LW Treatment of rheumatoid arthritis with anakinra, a recombinant human interleukin-1 receptor antagonist, in combination with methotrexate: results of a twenty-four-week, multicenter, randomized, double-blind, placebo-controlled trial. Arthritis Rheum (2002) 46(3):614–24 10.1002/art.10103 11920396

[32] Fleischmann RM Schechtman J Bennett R Handel ML Burmester GR Tesser J Anakinra, a recombinant human interleukin-1 receptor antagonist (r-metHuIL-1ra), in patients with rheumatoid arthritis: a large, international, multicenter, placebo-controlled trial. Arthritis Rheum (2003) 48(4):927–34 10.1002/art.10870 12687534

[33] Cohen SB Moreland LW Cush JJ Greenwald MW Block S Shergy WJ A multicentre, double blind, randomised, placebo controlled trial of anakinra (Kineret), a recombinant interleukin 1 receptor antagonist, in patients with rheumatoid arthritis treated with background methotrexate. Ann Rheum Dis (2004) 63(9):1062–8 10.1136/ard.2003.016014 15082469 PMC1755108

[34] Genovese MC Cohen S Moreland L Lium D Robbins S Newmark R Combination therapy with etanercept and anakinra in the treatment of patients with rheumatoid arthritis who have been treated unsuccessfully with methotrexate. Arthritis Rheum (2004) 50(5):1412–9 10.1002/art.20221 15146410

[35] Mertens M Singh JA Anakinra for rheumatoid arthritis. Cochrane Database Syst Rev (2009) 1:CD005121 10.1002/14651858.CD005121.pub3 19160248 PMC12296252

[36] Van den Broeck C Himpens E Vanhaesebrouck P Calders P Oostra A Influence of gestational age on the type of brain injury and neuromotor outcome in high-risk neonates. Eur J Pediatr (2008) 167(9):1005–9 10.1007/s00431-007-0629-2 18026751

[37] Schendel DE Schuchat A Thorsen P Public health issues related to infection in pregnancy and cerebral palsy. Ment Retard Dev Disabil Res Rev (2002) 8(1):39–45 10.1002/mrdd.10011 11921385

[38] Bracci R Buonocore G Chorioamnionitis: a risk factor for fetal and neonatal morbidity. Biol Neonate (2003) 83(2):85–96 10.1159/000067956 12576751

[39] McLean C Ferriero D Mechanisms of hypoxic-ischemic injury in the term infant. Semin Perinatol (2004) 28(6):425–32 10.1053/j.semperi.2004.10.005 15693399

[40] Boksa P Effects of prenatal infection on brain development and behavior: a review of findings from animal models. Brain Behav Immun (2010) 24(6):881–97 10.1016/j.bbi.2010.03.005 20230889

[41] Arrode-Bruses G Bruses JL Maternal immune activation by poly I:C induces expression of cytokines IL-1beta and IL-13, chemokine MCP-1 and colony stimulating factor VEGF in fetal mouse brain. J Neuroinflammation (2012) 9:83 10.1186/1742-2094-9-83 22546005 PMC3413576

[42] Hagberg H Gressens P Mallard C Inflammation during fetal and neonatal life: implications for neurologic and neuropsychiatric disease in children and adults. Ann Neurol (2012) 71(4):444–57 10.1002/ana.22620 22334391

[43] Meyer U Developmental neuroinflammation and schizophrenia. Prog Neuropsychopharmacol Biol Psychiatry (2013) 42:20–34 10.1016/j.pnpbp.2011.11.003 22122877

[44] Allan SM Pinteaux E The interleukin-1 system: an attractive and viable therapeutic target in neurodegenerative disease. Curr Drug Targets CNS Neurol Disord (2003) 2(5):293–302 10.2174/1568007033482742 14529361

[45] Rothwell N Interleukin-1 and neuronal injury: mechanisms, modification, and therapeutic potential. Brain Behav Immun (2003) 17(3):152–7 10.1016/S0889-1591(02)00098-3 12706413

[46] Cai Z Lin S Pang Y Rhodes PG Brain injury induced by intracerebral injection of interleukin-1beta and tumor necrosis factor-alpha in the neonatal rat. Pediatr Res (2004) 56(3):377–84 10.1203/01.PDR.0000134249.92944.14 15201401

[47] Allan SM Tyrrell PJ Rothwell NJ Interleukin-1 and neuronal injury. Nat Rev Immunol (2005) 5(8):629–40 10.1038/nri1664 16034365

[48] Thornton P Pinteaux E Gibson RM Allan SM Rothwell NJ Interleukin-1-induced neurotoxicity is mediated by glia and requires caspase activation and free radical release. J Neurochem (2006) 98(1):258–66 10.1111/j.1471-4159.2006.03872.x 16805812

[49] Denes A Pinteaux E Rothwell NJ Allan SM Interleukin-1 and stroke: biomarker, harbinger of damage, and therapeutic target. Cerebrovasc Dis (2011) 32(6):517–27 10.1159/000332205 22104408

[50] Savard A Lavoie K Brochu ME Grbic D Lepage M Gris D Involvement of neuronal IL-1beta in acquired brain lesions in a rat model of neonatal encephalopathy. J Neuroinflammation (2013) 10:110 10.1186/1742-2094-10-110 24007297 PMC3844447

[51] Green HF Treacy E Keohane AK Sullivan AM O’Keeffe GW Nolan YM A role for interleukin-1beta in determining the lineage fate of embryonic rat hippocampal neural precursor cells. Mol Cell Neurosci (2012) 49(3):311–21 10.1016/j.mcn.2012.01.001 22270046

[52] Fatemi SH Emamian ES Sidwell RW Kist DA Stary JM Earle JA Human influenza viral infection in utero alters glial fibrillary acidic protein immunoreactivity in the developing brains of neonatal mice. Mol Psychiatry (2002) 7(6):633–40 10.1038/sj.mp.4001046 12140787

[53] Aden U Favrais G Plaisant F Winerdal M Felderhoff-Mueser U Lampa J Systemic inflammation sensitizes the neonatal brain to excitotoxicity through a pro-/anti-inflammatory imbalance: key role of TNFalpha pathway and protection by etanercept. Brain Behav Immun (2010) 24(5):747–58 10.1016/j.bbi.2009.10.010 19861157

[54] Chang EY Zhang J Sullivan S Newman R Singh I N-acetylcysteine attenuates the maternal and fetal proinflammatory response to intrauterine LPS injection in an animal model for preterm birth and brain injury. J Matern Fetal Neonatal Med (2011) 24(5):732–40 10.3109/14767058.2010.528089 21219105

[55] Burd I Balakrishnan B Kannan S Models of fetal brain injury, intrauterine inflammation, and preterm birth. Am J Reprod Immunol (2012) 67(4):287–94 10.1111/j.1600-0897.2012.01110.x 22380481

[56] Dada T Rosenzweig JM Al Shammary M Firdaus W Al Rebh S Borbiev T Mouse model of intrauterine inflammation: sex-specific differences in long-term neurologic and immune sequelae. Brain Behav Immun (2014) 38:142–50 10.1016/j.bbi.2014.01.014 24486323 PMC3989501

[57] Bell MJ Hallenbeck JM Effects of intrauterine inflammation on developing rat brain. J Neurosci Res (2002) 70(4):570–9 10.1002/jnr.10423 12404511

[58] Auvin S Shin D Mazarati A Nakagawa J Miyamoto J Sankar R Inflammation exacerbates seizure-induced injury in the immature brain. Epilepsia (2007) 48(Suppl 5):27–34 10.1111/j.1528-1167.2007.01239.x 17910578

[59] Beloosesky R Ginsberg Y Khatib N Maravi N Ross MG Itskovitz-Eldor J Prophylactic maternal N-acetylcysteine in rats prevents maternal inflammation-induced offspring cerebral injury shown on magnetic resonance imaging. Am J Obstet Gynecol (2013) 208(3):e211–6 10.1016/j.ajog.2013.01.023 23433325

[60] Hagberg H Peebles D Mallard C Models of white matter injury: comparison of infectious, hypoxic-ischemic, and excitotoxic insults. Ment Retard Dev Disabil Res Rev (2002) 8(1):30–8 10.1002/mrdd.10020 11921384

[61] Kannan S Saadani-Makki F Muzik O Chakraborty P Mangner TJ Janisse J Microglial activation in perinatal rabbit brain induced by intrauterine inflammation: detection with 11C-(R)-PK11195 and small-animal PET. J Nucl Med (2007) 48(6):946–54 10.2967/jnumed.106.038539 17504871

[62] Young RS Hernandez MJ Yagel SK Selective reduction of blood flow to white matter during hypotension in newborn dogs: a possible mechanism of periventricular leukomalacia. Ann Neurol (1982) 12(5):445–8 10.1002/ana.410120506 7181450

[63] Yawno T Schuilwerve J Moss TJ Vosdoganes P Westover AJ Afandi E Human amnion epithelial cells reduce fetal brain injury in response to intrauterine inflammation. Dev Neurosci (2013) 35(2–3):272–82 10.1159/000346683 23571644

[64] Debillon T Gras-Leguen C Verielle V Winer N Caillon J Roze JC Intrauterine infection induces programmed cell death in rabbit periventricular white matter. Pediatr Res (2000) 47(6):736–42 10.1203/00006450-200006000-00009 10832730

[65] Fan LW Tien LT Zheng B Pang Y Rhodes PG Cai Z Interleukin-1beta-induced brain injury and neurobehavioral dysfunctions in juvenile rats can be attenuated by alpha-phenyl-n-tert-butyl-nitrone. Neuroscience (2010) 168(1):240–52 10.1016/j.neuroscience.2010.03.024 20346393 PMC2873102

[66] Shi L Fatemi SH Sidwell RW Patterson PH Maternal influenza infection causes marked behavioral and pharmacological changes in the offspring. J Neurosci (2003) 23(1):297–302 12514227 10.1523/JNEUROSCI.23-01-00297.2003 PMC6742135

[67] Meyer U Feldon J Schedlowski M Yee BK Immunological stress at the maternal-foetal interface: a link between neurodevelopment and adult psychopathology. Brain Behav Immun (2006) 20(4):378–88 10.1016/j.bbi.2005.11.003 16378711

[68] Cai Z Pan ZL Pang Y Evans OB Rhodes PG Cytokine induction in fetal rat brains and brain injury in neonatal rats after maternal lipopolysaccharide administration. Pediatr Res (2000) 47(1):64–72 10.1203/00006450-200001000-00013 10625084

[69] Burd I Brown A Gonzalez JM Chai J Elovitz MA A mouse model of term chorioamnionitis: unraveling causes of adverse neurological outcomes. Reprod Sci (2011) 18(9):900–7 10.1177/1933719111398498 21421895 PMC3343123

[70] Cai Z Fan LW Lin S Pang Y Rhodes PG Intranasal administration of insulin-like growth factor-1 protects against lipopolysaccharide-induced injury in the developing rat brain. Neuroscience (2011) 194:195–207 10.1016/j.neuroscience.2011.08.003 21840378 PMC3183292

[71] Hagan P Barks JD Yabut M Davidson BL Roessler B Silverstein FS Adenovirus-mediated over-expression of interleukin-1 receptor antagonist reduces susceptibility to excitotoxic brain injury in perinatal rats. Neuroscience (1996) 75(4):1033–45 10.1016/0306-4522(96)00225-4 8938739

[72] Ide CF Scripter JL Coltman BW Dotson RS Snyder DC Jelaso A Cellular and molecular correlates to plasticity during recovery from injury in the developing mammalian brain. Prog Brain Res (1996) 108:365–77 10.1016/S0079-6123(08)62552-2 8979814

[73] Scripter JL Ko J Kow K Arimura A Ide CF Regulation by interleukin-1beta of formation of a line of delimiting astrocytes following prenatal trauma to the brain of the mouse. Exp Neurol (1997) 145(2 Pt 1):329–41 10.1006/exnr.1997.6470 9217070

[74] Yoon BH Romero R Jun JK Park KH Park JD Ghezzi F Amniotic fluid cytokines (interleukin-6, tumor necrosis factor-alpha, interleukin-1 beta, and interleukin-8) and the risk for the development of bronchopulmonary dysplasia. Am J Obstet Gynecol (1997) 177(4):825–30 10.1016/S0002-9378(97)70276-X 9369827

[75] Kadhim H Tabarki B De Prez C Sebire G Cytokine immunoreactivity in cortical and subcortical neurons in periventricular leukomalacia: are cytokines implicated in neuronal dysfunction in cerebral palsy? Acta Neuropathol (2003) 105(3):209–16 10.1007/s00401-002-0633-6 12557006

[76] Girard S Sebire G Kadhim H Proinflammatory orientation of the interleukin 1 system and downstream induction of matrix metalloproteinase 9 in the pathophysiology of human perinatal white matter damage. J Neuropathol Exp Neurol (2010) 69(11):1116–29 10.1097/NEN.0b013e3181f971e4 20940629

[77] Rice JE III Vannucci RC Brierley JB The influence of immaturity on hypoxic-ischemic brain damage in the rat. Ann Neurol (1981) 9(2):131–41 10.1002/ana.410090206 7235629

[78] Comi AM Johnston MV Wilson MA Immature mouse unilateral carotid ligation model of stroke. J Child Neurol (2005) 20(12):980–3 10.1177/08830738050200120901 16417846

[79] Johnston MV Ferriero DM Vannucci SJ Hagberg H Models of cerebral palsy: which ones are best? J Child Neurol (2005) 20(12):984–7 10.1177/08830738050200091101 16417847

[80] Falahati S Breu M Waickman AT Phillips AW Arauz EJ Snyder S Ischemia-induced neuroinflammation is associated with disrupted development of oligodendrocyte progenitors in a model of periventricular leukomalacia. Dev Neurosci (2013) 35(2–3):182–96 10.1159/000346682 23445614 PMC3764456

[81] Szaflarski J Burtrum D Silverstein FS Cerebral hypoxia-ischemia stimulates cytokine gene expression in perinatal rats. Stroke (1995) 26(6):1093–100 10.1161/01.STR.26.6.1093 7762028

[82] Silverstein FS Barks JD Hagan P Liu XH Ivacko J Szaflarski J Cytokines and perinatal brain injury. Neurochem Int (1997) 30(4–5):375–83 10.1016/S0197-0186(96)00072-1 9106251

[83] Carlsson Y Leverin AL Hedtjarn M Wang X Mallard C Hagberg H Role of mixed lineage kinase inhibition in neonatal hypoxia-ischemia. Dev Neurosci (2009) 31(5):420–6 10.1159/000232560 19672071

[84] Park EM Cho BP Volpe BT Cruz MO Joh TH Cho S Ibuprofen protects ischemia-induced neuronal injury via up-regulating interleukin-1 receptor antagonist expression. Neuroscience (2005) 132(3):625–31 10.1016/j.neuroscience.2005.01.021 15837124

[85] Quiniou C Kooli E Joyal JS Sapieha P Sennlaub F Lahaie I Interleukin-1 and ischemic brain injury in the newborn: development of a small molecule inhibitor of IL-1 receptor. Semin Perinatol (2008) 32(5):325–33 10.1053/j.semperi.2008.07.001 18929155

[86] Wang CH Wang WT Cheng SY Hung WT Wu TL Hsueh CM Leptin and interleukin-1beta modulate neuronal glutamate release and protect against glucose-oxygen-serum deprivation. Curr Neurovasc Res (2010) 7(3):223–37 10.2174/156720210791184925 20560877

[87] Martin D Chinookoswong N Miller G The interleukin-1 receptor antagonist (rhIL-1ra) protects against cerebral infarction in a rat model of hypoxia-ischemia. Exp Neurol (1994) 130(2):362–7 10.1006/exnr.1994.1215 7867766

[88] Hu X Nesic-Taylor O Qiu J Rea HC Fabian R Rassin DK Activation of nuclear factor-kappaB signaling pathway by interleukin-1 after hypoxia/ischemia in neonatal rat hippocampus and cortex. J Neurochem (2005) 93(1):26–37 10.1111/j.1471-4159.2004.02968.x 15773902

[89] Campion GV Lebsack ME Lookabaugh J Gordon G Catalano M Dose-range and dose-frequency study of recombinant human interleukin-1 receptor antagonist in patients with rheumatoid arthritis. The IL-1Ra Arthritis Study Group. Arthritis Rheum (1996) 39(7):1092–101 10.1002/art.1780390704 8670316

[90] Nuki G Bresnihan B Bear MB McCabe D Long-term safety and maintenance of clinical improvement following treatment with anakinra (recombinant human interleukin-1 receptor antagonist) in patients with rheumatoid arthritis: extension phase of a randomized, double-blind, placebo-controlled trial. Arthritis Rheum (2002) 46(11):2838–46 10.1002/art.10578 12428223

[91] Ferriero DM Neonatal brain injury. N Engl J Med (2004) 351(19):1985–95 10.1056/NEJMra041996 15525724

[92] Pierrat V Haouari N Liska A Thomas D Subtil D Truffert P Prevalence, causes, and outcome at 2 years of age of newborn encephalopathy: population based study. Arch Dis Child Fetal Neonatal Ed (2005) 90(3):F257–61 10.1136/adc.2003.047985 15846019 PMC1721886

[93] Graham EM Ruis KA Hartman AL Northington FJ Fox HE A systematic review of the role of intrapartum hypoxia-ischemia in the causation of neonatal encephalopathy. Am J Obstet Gynecol (2008) 199(6):587–95 10.1016/j.ajog.2008.06.094 19084096

[94] D’Alton ME Hankins GDV Berkowitz RL Bienstock J Ghidini A Goldsmith J Executive summary: neonatal encephalopathy and neurologic outcome, second edition. Report of the American College of Obstetricians and Gynecologists’ task force on neonatal encephalopathy. Obstet Gynecol (2014) 123(4):896–901 24785633 10.1097/01.AOG.0000445580.65983.d2

[95] Edwards AD Brocklehurst P Gunn AJ Halliday H Juszczak E Levene M Neurological outcomes at 18 months of age after moderate hypothermia for perinatal hypoxic ischaemic encephalopathy: synthesis and meta-analysis of trial data. BMJ (2010) 340:c363 10.1136/bmj.c363 20144981 PMC2819259

[96] Fan X van Bel F van der Kooij MA Heijnen CJ Groenendaal F Hypothermia and erythropoietin for neuroprotection after neonatal brain damage. Pediatr Res (2013) 73(1):18–23 10.1038/pr.2012.139 23085819

[97] Fang AY Gonzalez FF Sheldon RA Ferriero DM Effects of combination therapy using hypothermia and erythropoietin in a rat model of neonatal hypoxia-ischemia. Pediatr Res (2013) 73(1):12–7 10.1038/pr.2012.138 23085817 PMC3540182

